# Exploring medical students’ perceptions of empathy after cinemeducation based on Vygotsky’s theory

**DOI:** 10.1186/s12909-024-05084-z

**Published:** 2024-01-29

**Authors:** Mahla Salajegheh, Amir Ali Sohrabpour, Elaheh Mohammadi

**Affiliations:** 1 Department of Medical Education, Medical Education Development Center, Kerman University of Medical Sciences, Kerman, Iran; 2 Liver and Pancreatobiliary Diseases Research Center, Digestive Disease Research Institute, Tehran University of Medical Sciences, Tehran, Iran; 3 Health Professions Education Research Center, Educational Development Center, Tehran University of Medical Sciences, Tehran, Iran

**Keywords:** Empathy, Reflection, Medical students, Cinemeducation, Vygotsky's theory

## Abstract

**Background:**

Medical students’ empathy toward patients with Alzheimer’s is rarely found in formal medical curricula. Based on Vygotsky’s theory, watching films and reflection can be considered as effective methods to improve empathy. The present study aimed to explore medical students’ perceptions of empathy toward patients with Alzheimer after participating in an educational program by using interactive video based on Vygotsky’s theory.

**Methods:**

This qualitative study was conducted at Tehran University of Medical Sciences in 2022. The population included all 40 medical students. Firstly, the Still Alice movie which is about the feelings of a professor who was diagnosed with Alzheimer’s disease was shown to the students. Secondly, the students reflected on their experiences of watching the movie. Thirdly, a session was held for group discussion on the subject of the movie, the patient’s feelings, the doctor’s attitude, the social environment surrounding the patient shown in the movie, and the necessity of empathy toward patients with Alzheimer’s disease. The reflection papers were analyzed using the conventional qualitative content analysis method.

**Results:**

After analyzing 216 codes from 38 reflection papers, four categories, including communication with a patient with Alzheimer’s, understanding the patient with Alzheimer’s as a whole, medical science development, and the student’s individual ideology, were extracted.

**Conclusion:**

Reflection and group discussion after watching movie by providing opportunities for social interaction about personal interpretations will lead to active role in enhancing empathy. Based on the perceptions of the medical students, they gained a perspective to consider the patient as a whole and pay attention to establishing a proper relationship with the patient.

**Supplementary Information:**

The online version contains supplementary material available at 10.1186/s12909-024-05084-z.

## Introduction

One of the most vital aspects of a proper patient-physician relationship is empathy, which is described as the ability to understand and experience other people’s feelings and has moral, cognitive, emotional, and behavioral aspects [1, 2]. In the healthcare setting, empathy includes three parts: the sharing of a patient’s emotional state; the explicit comprehension of a patient’s emotional state; and the social behaviors that follow [3].

There is evidence that doctors who empathize have higher patient satisfaction, reduced malpractice claims, and higher levels of clinical competence [4]. In contrast, a lack of empathy was identified as a contributing factor to a culture of incompetence that threatened patient safety and confidence in the medical profession [5].

As a result, empathy’s important role in medical care is considered a basic skill for medical education [6]. To highlight its importance, the Association of American Medical Colleges argues that empathy should be a critical objective in undergraduate medical education. Thereafter in the curriculum of many medical schools around the world, empathy has been implemented in medical students’ training [7]. Despite the emphasis on empathy as a basic skill for physicians, in the curriculum, there is rarely a special educational program to teach and enhance empathy, and students may only learn this important factor in professional ethics when faced with the positive role models. Since it is valuable to observe the professional and empathetic behavior of role model faculty, but it may not happen to all students, it is important to have educational planned programs for improving empathy.

In general, the physician’s empathy with all patients is very important and effective in the treatment process. However, some diseases such as Alzheimer due to dementia may seem that the patient has less understanding and does not need empathy, so it may be that medical students do not pay attention to the need for empathy in these types of patients [8].

Alzheimer’s disease as a progressive neurologic disorder is the most common cause of dementia, and continuous cognitive and emotional deficits in patients [9]. In the context of patients with Alzheimer, it may feel that these patients do not need empathy and do not understand emotions [10]. But, several studies have examined emotions in patients with Alzheimer’s, and the results revealed that these patients have varying degrees of emotion at different stages of the disease [9]. Guzmán-Vélez et al. (2014) investigated feelings of emotion in patients with Alzheimer’s. The results indicated that these patients can experience prolonged states of emotion [11]. A study by Gomez-Gallego and Gomez-Garcia (2018) assessed the emotional experience of patients with Alzheimer’s and reported that these patients can identify the emotional content of the stimuli, and the emotional enhancement of memory is preserved [12].These findings highlight that although the emotional lives of patients with Alzheimer’s have significant implications for their care, educational programs for medical students about empathy toward these patients, are still relatively scarce [13–16]. Therefore, teaching students about empathy with all patients in general and emphasizing the need to empathize with patients with dementia is essential.

Different methods, including giving lectures in class, observing the empathic behavior, role-playing [17], studying patients’ narratives of their disease, books, poetry, and novels, watching photos, videos, sculptures, or listening to music [18], and analyzing audio or video records of patients encountering professional health cares [19] have been applied for improving empathy of medical students. Each of these methods, along with many strengths such as gaining a better insight and understanding of the patient’s condition, has some limitations that reduce the cultivating empathy in medical students.

Due to the multi-dimensional, complex, and subjective nature of empathy which includes both cognitive and affective learning domains, teaching methods relying on communication are recommended [20, 21]. This concept is in line with one of the social constructivist theories– i.e. Vygotsky’s theory.This theory emphasizes learning through collaboration with others, reflection and authentic activities based on real life situations [22] Therefore, one of the suitable methods for enhancing the empathy of medical students can be is watching relative movies about the emotional state of patients based on their real life [23]. Cinemeducation is a beneficial strategy in healthcare education especially for developing empathic engagement, which help to understand the difficulties, worries, feelings and emotions of the characters, experience a different reality, and extend this view to patients in the real environment [24]. Cinemeducation has been mentioned as an unique and enjoyable narrative medical approach to the teaching–learning of health humanities [25]. Cinemeducation in group settings would be helpful in brainstorming, creating useful ideas, and sharing perspectives about the scenes and characters in the movie from different perspectives. The cinemeducation can inspire students’ ability to reflect on their mental and professional actions in complex situations in protected settings [26].

Also, reflection on the experience can be an effective method to improve empathy [20]. Reflection lets the assimilation of concepts, skills, knowledge, and values into pre-existing knowledge structures and provides opportunities for medical students to clarify their opinions on observed behaviors [27].. The ability of cinemeducation to engage medical students in discussions about empathy toward patients with Alzheimer’s can be a part of the active learning process in Vygotsky’s Theory. Reflection and group discussion after watching the movie by providing suitable opportunities for social interaction about personal interpretations of the students’ experiences will lead to their active role in enhancing empathy [28]. The present study aimed to explore medical students’ perceptions of empathy toward patients with Alzheimer after participating in an educational program by using interactive video based on Vygotsky’s theory.

## Methodology

### Setting

This qualitative study was conducted at Tehran University of Medical Sciences (TUMS) in the internal department of Shariati hospital, a major hospital affiliated with TUMS. Clerkships have 2-week rotations and interns have 1-month rotations in the internal department. The research was carried on 2022. The study was approved by Health Professions Education Research Center (No IR.TUMS.MEDICINE.REC.1400.1088). Participants did not receive any incentives, and participation was voluntary. The participants were assured of the confidentiality of their information, and it was explained that the results would only be used for research objectives.

### Participants

The population included clerkships (*N* = 29) and interns (*N* = 11) of general medical students in internal rotation at TUMS. The majority of participants were male (*N* = 23- 57.5%). The inclusion criteria include being a student of general medicine at Tehran University of Medical Sciences, being clerkships or interns in internal rotations, and consenting to participate in the research. Exclusion criteria also include the student’s unwillingness to participate in research, non-participation in the movie-showing session or group discussion, and not completing the reflection paper. Finally, 38 paper reflections were analysed.

### Intervention

In this study, interactive video method was used to teach empathy to students. The use of watching movie to create an insight of empathy with patients and their families along with reflection and group discussion can help to improve the insight of empathy in students and have more long-term impacts. Therefore, the intervention included three phases. Firstly, the Still Alice movie was shown to the participants in the conference hall of the hospital. The movie stories the feelings and experiences of a linguistics professor who was diagnosed with Alzheimer’s disease. Watching this movie, which deals with the different aspects of the life of a person with Alzheimer’s disease, can help to understand the life of these patients and their personal and family problems, and lead to the promotion of awareness and empathic insight with them.

Reflection on what was watched in the movie about the patients with Alzheimer can help to strengthen and perpetuate the findings from watching the movie in students. So, in addition to drawing personal conclusions, they can prepare for real situations of encountering these patients as a physician. Therefore, in the second phase, at first students were taught how to do reflection. Then, the students were asked to reflect on their experiences of watching the movie through reflection forms, within two days. The reflection forms, including five open-ended questions, were prepared based on Gibbs’ Reflective Cycle. The Gibbs model is one of the cyclical models of reflection, which helps in effective reflection by performing steps. According to this model, individuals first describes their experience and expresses feelings in that field, and then, by evaluating and analyzing their strengths and weaknesses, plans to improve performance in the future [29]. (A copy of the reflection form is included in Supplementary 1). Some examples of the questions in the reflection forms are as follows:

- In your opinion (as a future doctor), what are the effects of watching this movie on better understanding the lives of patient with Alzheimer disease and their problems?
- What changes in your attitude towards the patient with Alzheimer disease occurred after watching this movie?
- As a future doctor, what effects do you think watching this movie will have on your future dealings with a patient with Alzheimer disease?

Thirdly, another session was held two days later. The students gave the completed reflection forms to the researchers. Then, the researchers facilitated a group discussion among the students about the subject of the movie, the patient’s feelings, the doctor’s attitude, the social environment surrounding the patient shown in the movie, and the necessity of empathy toward patients with Alzheimer’s disease. According to Vygotsky’s theory, interactive discussion helps to build knowledge in students. This session lasted for approximately 90 min. The researchers ensured that all participants were active in the group discussion in a fair and constructive way.

### Method within the context of Vygotsky theory

Constructivism has a strong impact on the learning process of the multi-dimensional, complex, and subjective concepts as a dominant education philosophy. The learning approaches and teaching methods based on constructivism are influenced by the theories of Piaget and Vygotsky [30]. In dynamic learning methods created based on constructivist theories, the learner plays the role of the constructor of information and takes an active character [31]. They must perform meaningful learning activities and think about what they are doing in an active learning process [32]. Vygotsky’s theory highlighted the reality that the learning process depends on collaborations with others and the significance of the reflection of this interaction process to an individual’s internal world [30].

In the present research, in accordance with this theory, knowledge transfer was done through watching movie and with interactive activities in an active learning environment. By reflection and participation in a group discussion, the students were actively involved in the learning process in a cooperative manner. In fact, they shared the knowledge they had obtained with other students and during the group discussion session, they actively had the role of structuring information.

Vygotsky stressed two main points in the learning process including culture and language [31]. In the present method, a suitable environment was actively provided for the transfer of culture, social structures and values to students through watching movie and group discussions.

According to Vygotsky, language is enhanced with social relations that is critical for cognitive development [33]. As a result of the person’s social communications, their talks with others and with themselves privately are more related to cooperative learning, so they were mentioned in this study.

Vygotsky also claims the principle of Zone of Proximal Development (ZPD) which emphasized that the learning process achieve more effectively as a result of involvement of students with peers that are more informed or their teachers [30].

In the present method, students from two different groups including clerkships and interns discussed in a group about the watched movie and the reflections. The group discussion was led by two faculty members as facilitators. Therefore, in this collaborative learning, through the social interactions of peers with different levels and experiences and the guidance of the facilitators, the principle of the ZPD of Vygotsky’s theory was realized.

According to Vygotsky, the process of teaching and learning must be comprehended via authentic activities which means be based on real-life situations and must be meaningful to the students [31].

In the present method, the students watched the movie which shows the life of a person with Alzheimer in the face of the real-life problems. So, the students could put themselves in the place of the movie character and understand her real life in different dimensions.

### Data analysis

Data were analyzed using the conventional qualitative content analysis method [34]. The reflection papers were read several times by (EM) for better understanding and general comprehension. Then, each reflection paper was divided into meaning units. The meaning units were coded and the codes were divided into sub-categories and categories based on similarities and differences.

### Rigor

Each of the analytic reports was reviewed in terms of reflecting the viewpoints of students. The research team reflected on the findings of the study and reached a consensus. A summary of the reflection papers was returned to the students as a member check and approved by them [34]. A peer check was also performed by one author, who was familiar with the qualitative content analysis method. To confirm dependability, several reflection papers were randomly analyzed by an audit to ensure the accuracy of the process. The plausibility of the findings confirmed that the analyses and interpretations were justifiable.

## Results

In the present study, participants have explored their perceptions of empathy toward patients with Alzheimer’s after participating in an interactive educational program. The results of the qualitative content analysis of 216 codes from 38 reflection papers can be presented in four categories, including communication with a patient with Alzheimer’s, understanding the patient with Alzheimer’s as a whole, medical science development, and the student’s individual ideology.

### Communication with a patient with Alzheimer’s

According to the participants, professional patient-physician communication was one of the components of empathy toward patients with Alzheimer’s. They believed that it could be very influential in the treatment of these patients and includes communication with patients with Alzheimer’s, communication with the families of these patients, and mental and psychological support of these patients.

1. Communication with the patient with Alzheimer’s.

Some participants supposed that a part of empathy toward patients with Alzheimer’s was to effectively communicate with the patient. As shared by P42, “I will try to treat patients with Alzheimer’s respectfully, because this disease is a shocking crisis. In order not to hurt their feelings, I deal with them more carefully.”

2. Communication with the family of the patient with Alzheimer’s.

Another finding regarding empathy toward patients with Alzheimer’s was related to the efficient communication with the families of these patients that developed productivity as described in some reflections. As noted by P6, “This disease is not well known and causes a lot of fear for the person and those around him, and we, as doctors, are responsible for the peace of their mind, making the family members aware of this disease, explaining the upcoming events, and showing enough patience in dealing with the patient’s family members who bear a lot of pressure.”

3. Mental and psychological support of the patient with Alzheimer’s.

Some of the participants believed that their willingness for the mental and psychological support of these patients increased. “Now I better understand the effects of this disease on various aspects of the personal, family, and social lives of these patients and I will try to provide the best support for these patients and improve their quality of life.” (P14) and “Sometimes it is necessary to have psychiatric treatment, we can request counseling and refer to the relevant experts.” (P2).

### Understanding the patient with Alzheimer’s as a whole

One of the components of being a proper physician is providing holistic care involves treating a patient as a “whole” person instead of focusing on just the illness. Some students described their attitudes toward the patient with Alzheimer’s in terms of increasing awareness and understanding of their emotional, physical, and social conditions after participating in the interactive educational program.

1. Understanding the emotional conditions of the patient with Alzheimer’s

Living with Alzheimer’s disease affects a person’s feelings, thoughts, and responses and leads to a range of symptoms such as depression, aggression, anxiety, and agitation. In the treatment process, it is significant for the physician to know and respond to the patient’s emotional needs. As P26 described, “Any medical book can’t help us to understand their mental and emotional states, but watching this movie made me put myself into the patient’s shoes and understand his condition better”, this interactive educational program improves their awareness of the emotional conditions of the patient with Alzheimer’s.

2. Understanding the physical conditions of the patient with Alzheimer

As Alzheimer’s disease progresses to its last stages, brain changes begin to affect physical functions, such as swallowing, balance, and bowel and bladder control. Some participants believed that they gain a perspective on the physical conditions of the patient with Alzheimer’s. “Before this, I had never put myself in the patient’s place. I had not seen what difficult moments they were going through and what physical problems they were suffering from.” (P11).

3. Understanding the social conditions of the patient with Alzheimer

Before the disease, many patients with Alzheimer’s disease were useful and effective people in society, and probably they were in high social and occupational positions, but after the disease, they were not able to continue their social activities. Some students believed that participation in this program has helped them to better understand the social conditions of the patients, and they can imagine that every patient might be a doctor, a teacher, or… so they will treat him with more respect. “Many of them were important and useful people, and might provide a lot of services to society. Now, the current situation is very difficult for them and their families to bear.” (P6).

4. Changing the attitude toward the patient with Alzheimer’s

A physician’s positive attitude toward the patient with Alzheimer’s is an important factor in early diagnosis of the disease, timely intervention, and treatment. According to the participants’ perceptions of the patient with Alzheimer’s, strengthening attitudes to support these patients and provide better healthcare was emphasized. “Until now, I didn’t know how painful and difficult everything can be for these people. As doctors, most of the symptoms related to the diagnosis of a disease are valuable to us… but it is very important to pay attention to the fact that the disease is not just a series of ordinary symptoms and affects the present and future of the patient and those around him.” (P11).

### Medical science development

According to the participants, watching the movie and participating in this program, have helped them in developing medical knowledge in the field of Alzheimer’s disease, increased their awareness and medical knowledge of better treatment of Alzheimer’s disease, and even motivated them to perform research on Alzheimer’s disease.

1. Helping the doctor perform better in the field of treatment and encouraging scientific research about Alzheimer’s disease.

Some students reported that by participating in this program, they will perform better in providing treatment to patients with Alzheimer’s. Some believed that they would prefer to conduct research in the field of new treatments and new ways to manage Alzheimer’s disease. “By watching this movie and knowing the symptoms better, I will get a more appropriate history from these patients. I try to advise my patients as much as possible about the ways to postpone each side effect. I want to do more research in this field.” (P32) and “By watching this movie, I learned how to deliver the news of this disease to the patients and their families.” (P15).

2. Desire to promote knowledge of Alzheimer’s disease.

Many students believed that with this educational experience, their knowledge of Alzheimer’s disease, its symptoms, and treatment has increased. “Watching this kind of movie helps to understand the conditions and sufferings of patients, it also helps to learn the early and late symptoms of the disease, and we can remember them better. It is also effective in diagnosis and treatment of the disease.” (P38).

### The student’s individual ideology

It was found that the cognitive orientation of students, including philosophy, norms, values, emotions, and ethics, has a significant effect on their empathy toward patients with Alzheimer’s. This main category contains two sub-categories including the student’s attitude toward life and arousing the feeling of fear and discomfort.

1. Student’s attitude toward life.

Doctors have different attitudes and beliefs toward patients with Alzheimer’s, which affect their behavior in providing health services to these patients. Some students considered the impact of the instability of life, destiny, the unpredictability of the future, the value of life, and human identity on their empathy toward patients with Alzheimer’s. “It was a good experience. Full of thinking about the purpose of life… the identity of a person and her values” (P25) and “The impermanence and the power of change in life that turns this scene. What matters is that we live” (P17).

2. Arousing the feeling of fear and discomfort.

Some of the students believed that after watching this movie, they had feelings and worries such as the fear that they or their families will get Alzheimer’s disease, and they felt sadness for patients with Alzheimer’s. “The feeling of fear took over my whole being, first concerning my family, grandparents, then my parents, if this happens to them, how can I cope with it,? Is it possible that one day I will forget that a movie about Alzheimer’s causes this fear in me or not?!” (P22).

## Discussion

Considering the importance of empathy of future physicians toward patients with Alzheimer’s, exploring the perceptions of medical students on empathy toward patients with Alzheimer’s after participating in an interactive educational program can be helpful for medical educators to foster empathy. This study provided a unique opportunity for medical students to collaborate during an interactive educational program including watching a movie, self-reflection, and group discussion. Our study is the first, to our knowledge, to provide this valuable experience for medical students. The study derived four categories of perceptions of medical students on empathy toward patients with Alzheimer’s including communication with a patient with Alzheimer, understanding the patient with Alzheimer’s as a whole, medical science development, and the student’s individual ideology.

Effective communication with patients with dementia is very important. In this regard, patients with Alzheimer’s, because of the special symptoms of this disease, such as depression, anxiety, agitation, and so on, need more consideration [35]. Indeed, these patients often, due to dementia, are assumed to not understand emotions and to not need empathy [10]. There is evidence that, even in the advanced stages of Alzheimer’s disease, there is a significant emotional need for communication [36]. During the progression of Alzheimer’s disease, effective communication has a great impact on the treatment process but also is an effective factor in their quality of life [37]. Therefore, communication needs to be a part of life for people with Alzheimer’s. This subject emphasizes the vital need to improve the communication of future physicians with these patients. So far, limited studies have addressed the communication and its aspects as essential components of good quality care of patients with Alzheimer’s. Bickford et al. (2019) explored the understanding of undergraduate medical and nursing students of compassion towards people with dementia. One of their emergent themes was connection which was identified as an awareness and understanding of the person behind the diagnosis [38]. Campbell et al. (2021) described and reflected on the experience of future nurses in a simulated virtual reality dementia program and noted that improved communication with patients with Alzheimer’s disease was one of the main outcomes. Some of their participants believed that they achieved the ability to understand the stress that the families of patients with Alzheimer’s experience [39]. The present results acknowledge the need to support the families of patients with Alzheimer’s disease. Moreover, it is worth mentioning that the manifestation of empathy arises from the communication between a physician and a patient with Alzheimer’s disease. In a clinical education setting, this phenomenon occurs in front of watchful eyes of students, highlighting the importance of the role modeling of clinical teachers [40]. Clinical educators should emphasize the interpersonal nature of empathy, and encourage students to be genuinely interested to make suitable relationships with patients with Alzheimer’s disease.

Based on the medical students’ experiences, they developed the perspective of seeing the patient as a person throughout training. This finding emphasizes the importance of preparing future physicians for holistic healthcare [41], which is stated in the experiences of our medical students comprehensively. An important outcome of our educational program was that participants begin to thoughtfully consider the physical, emotional, and social needs of a patient with Alzheimer’s disease. Krishnasamy et al. (2019) conducted a meta-ethnography to explore an understanding of how medical education affects the expression of empathy and compassion in medical students. They derived four main themes one of which was seeing the patient as a person. This result is similar to our results [28]. This finding was also reported in a study by Slater et al. (2019) who conducted qualitative exploratory research to explore the impact of an interactive training experience on moral, emotive, behavioral, and cognitive elements of care of people with dementia. They recognized patient care holistically as a behavioral response among participants [42].

Our results echo the recommendation to develop holistic healthcare through educational activities incorporating relations with patients, along with narrative approaches, such as self-reflection and videos [43]. The provision of these opportunities allowed medical students to clarify and relate the observed behaviors of patients with Alzheimer’s disease to future learning experiences of empathy.

Dementia education has been confirmed of enhancing Alzheimer’s disease knowledge, especially for healthcare providers [44]. Chan et al. (2022) assessed the knowledge of dementia among final-year medical undergraduates across 7 universities in Malaysia. The results of this study showed that overall dementia knowledge was low, especially in communication with these patients. They revealed that higher knowledge of dementia was associated with informal education and occupational/working experience [45], which are consistent with our findings. Our results suggest that medical curricula should have revised to incorporate informal education to enhance knowledge about Alzheimer’s disease in future physicians.

An interesting finding of our study is the effect of medical students’ attitudes and beliefs toward Alzheimer’s disease. Some felt worries and anxiety about getting Alzheimer’s themselves or their families in the future. Some previous studies also reported depression, fear, sleeplessness, and stress in healthcare providers [46]. This finding highlights the high importance of physicians and medical students’ mental health, who are exposed to patients with Alzheimer’s disease and their need to actively adopt psychological services.

The findings of our study are important, they highlight that the experience of cinemeducation, self-reflection and group discussion after it provides a different way of learning empathetic behaviors. These results emphasize the findings of previous studies about cinemeducation which used movies to address various topics of professionalism and ethics such as empathy. Patel et al. (2022) taught professionalism and ethics to undergraduate students using cinemeducation and evaluated the impact by questionnaire, role-playing, and reflection. They revealed that cinemeducation is an effective training tool to understand patients’ emotions, the important role of communication, and to improve empathy in patient care [47]. Due to the multi-dimensional, complex, and subjective nature of empathy, movies convey this difficult concept in real-life situations for students and stimulate reflection and discussion. Therefore, cinemeducation is a beneficial and also interesting teaching method for helping medical students to promote the development of empathy with patients. Our findings highlighted that cinemeducation may contribute to enabling health professionals to improve empathetic and relational skills.

Discussion is an effective teaching and learning method. The past studies have discovered that the quality of the discussions may differ based on what educational activity comes before them [48]. The results echo that the quality of the discussions improved when watching the movie and self-reflection preceded them due to the positive effect active learning had on enhancing empathy.

Our findings highlighted important curricular gaps within the general medicine curricula regarding the teaching of empathy toward patients with Alzheimer’s disease. These also provide support for enhancing experiential learning opportunities for medical students related to Alzheimer’s disease.

### Limitations

There was lack of maximum variation sampling in our study. In addition, there was also lack of triangulation method in this study. Using of triangulation method can develop a comprehensive understanding of phenomena. Further studies would benefit from employing different methodologies especially mixed-methods approaches and quantitative studies to seek additional evidence of the effectiveness of innovative strategies on the empathetic behaviors of students through patients with Alzheimer’s disease. Furthermore, it would have been better if the educational intervention had been held as a long-time program and several movies on the subject studied would have been shown. Also, the use of interviews, in addition to reflection papers, could provide more reliable results.

### Recommendations for future studies

More exploration is needed to create how improved empathy may be further applied to improve care for patients with Alzheimer’s disease. In addition, to enrich the content, it is recommended to use other data collection methods such as interviews along with reflection. Also, we recommend that future studies with a suitable sample size be conducted to investigate the quantitative effectiveness of the educational method through intervention and comparison with the control group.

## Conclusion

An interactive educational program, including watching a movie, self-reflection, and group discussion experience, is verified to be a valuable strategy to enhance medical students’ empathy toward patients with Alzheimer’s disease. According to the perceptions of medical students, after participating in the educational program, their attitudes towards patients with Alzheimer changed, and the communication with a patient with Alzheimer’s and understanding of him/her as a whole were enhanced regarding the special needs of these patients.

### Electronic supplementary material

Below is the link to the electronic supplementary material.

Supplementary Material 1

## Data Availability

The datasets used and/or analyzed during the current study are available from the corresponding author on reasonable request.
