# Supplementary material for: Exploring medical students’ perceptions of empathy after cinemeducation based on Vygotsky’s theory
Source: BMC Med Educ. 2024 Jan 29;24:94. doi: 10.1186/s12909-024-05084-z (PMC10823714; doi:10.1186/s12909-024-05084-z)
Supplement: Supplementary file 1 — Supplementary Material 1 [file 12909_2024_5084_MOESM1_ESM.docx]

**A copy of the reflection form**

| The student reflection form  Name of student:  Date and time:  Please describe your experience of watching the Still Alice movie,   - How did you feel during watching this movie about the life of a patient with Alzheimer's disease? - What scene or point of the movie was interesting to you? Why? - In your opinion (as a future doctor), what are the effects of watching this movie on better understanding the lives of the patients with Alzheimer's disease and their problems? - What changes in your attitude towards the patient with Alzheimer's disease occurred after watching this movie? - As a future doctor, what effects do you think watching this movie will have on your future dealings with a patient with Alzheimer's disease? |
| --- |
